# VacA’s Induction of VacA-Containing Vacuoles (VCVs) and Their Immunomodulatory Activities on Human T Cells

**DOI:** 10.3390/toxins8060190

**Published:** 2016-06-18

**Authors:** Ciara Utsch, Rainer Haas

**Affiliations:** Max von Pettenkofer-Institut für Hygiene und Medizinische Mikrobiologie, Ludwig-Maximilians-Universität, Pettenkoferstraße 9a, München D-80336, Germany

**Keywords:** cell vacuolation, VacA channel, protein-protein interaction, apoptosis, IL-2 secretion, proliferation inhibition, vesicle transport

## Abstract

Vacuolating cytotoxin A (VacA) is a secreted pore-forming toxin and one of the major virulence factors of *Helicobacter pylori* (*H. pylori*), which actively supports the persistence and survival of the bacteria in the special ecological niche of the human stomach. *H. pylori* genomes harbor different allelic forms of the *vacA* gene, which translate into functionally distinct VacA toxin types. VacA internalizes into various cell types via membrane or specific receptor interactions finally forming acidic endocytic VacA-containing vacuoles (VCVs). In this review, we focus on different characteristics of VacA, its interaction with host cells, the formation and protein content of VCVs and their intracellular transport into human T cells, which finally leads to the immunosuppressive phenotype of VacA. Immunomodulatory activities of VacA on human T cells are discussed with a focus on T-cell proliferation and calcium signaling.

## 1. Introduction

The human bacterial pathogen *Helicobacter pylori (H. pylori)*, first cultured and identified in 1982, still infects more than 50% of the world’s population [1]. *H. pylori* contributes to the development of diseases such as gastritis, duodenal and gastric ulcers, and gastric cancer, earning it a place on the WHO list of class 1 carcinogens, even though only a minority of infected individuals develop clinical symptoms [2]. It holds an impressive inventory of virulence factors, which enable the bacteria to persist in the human stomach and cause lifelong infections. Among these, the vacuolating cytotoxin A (VacA) displays an impressive list of abilities [3]. VacA has been termed a paradigm for toxin multifunctionality [3]. Research on VacA over the last quarter of a century reveals the impressive association of this toxin with many different cell types, affecting membrane structure and function, the endoplasmic reticulum (ER), the mitochondria, and the Golgi apparatus, seemingly causing a plethora of cellular effects, including the concurrent suppression and stimulation of immune cells among other things [4,5]. Its main defining feature is the formation of large acidic intracellular vacuoles [6]. This review will briefly outline VacA’s composition and some of its effects before delving further into the possible intracellular movements of the VacA-containing vacuoles (VCVs) and their effects on human T cells, especially its immunomodulatory potential. 

## 2. The Production of Functionally Distinct VacA Toxin Types by *H. pylori*

The *vacA* gene codes for a signaling sequence, the p33, p55, and autotransporter domains and translates into a 140-kDa protoxin [3,4,7]. Mosaicism of this gene has been found to influence the toxicity, namely variations in the signal sequence (s1a-c, s2), the intermediate region (i1, i2), and the mid-region (m1, m2) [8]. From early on, varying disease probabilities have been linked to the different s and m regions of VacA. Thus, most effective immune regulatory effects and an association with generally more severe disease states have been attributed to an s1/m1 genotype [8,9]. Gonzalez-Rivera *et al.* showed that an i1 form of VacA was more adept at inhibiting T-cell function as well as binding better to the target T cells than an i2 form [10]. Winter *et al.* discovered a correlation between the presence of the i1 form of VacA and gastric metaplasia in patients, while the i2 form of VacA was associated with an almost complete absence of metaplasia in the stomach of *H. pylori*-infected individuals [11]. To become active, the protoxin is processed at the amino- and carboxyl-terminals, yielding a mature 88-kDa cytotoxin, which is then secreted via a type Va secretion system [7,12]. As VacA is secreted it forms spontaneous hexameric and dodecameric flower-shaped structures [13]. These oligomers need to be monomerized via acid-activation to be internalized and to fully unfold their potential [13]. The p33 *N*-terminal region contains a short residue sequence essential for vacuolating activities [14], and the p55 *C*-terminal region is important for the toxins’ target binding abilities. However, both fragments are needed for internalization and vacuolation [15]. Near the amino-terminal, there is a short and very hydrophobic segment, whose presence is required for vacuole formation. Bacterial mutants missing a 21 amino acid stretch (Δ6-27aa) of this 32 amino acid region produce a VacA toxin that is incapable of causing vacuolation [14,16]. 

## 3. The Effects of VacA on Eukaryotic Cells

Several distinct effects of the VacA toxin have been described in different cell types *in vitro* and the gastric epithelium *in vivo*. The occurrence of vacuolation in cells incubated with *H. pylori* culture supernatant was the first piece of evidence of a secreted toxin being responsible for some of the cellular damage possibly causing ulcers [6,17]. Since that discovery, many more effects have been added to the list of abilities of the vacuolating toxin A. Remarkably, it causes permeabilization of the plasma membrane [18], reduction of the mitochondrial transmembrane potential, mitochondrial cytochrome C release [19,20,21], mitochondrial fragmentation [22], and even cell cycle arrest [14]. Furthermore, VacA is known to cause cell death via apoptosis [23,24] as well as programmed necrosis [25]. Initially, VacA was studied exclusively for its effects on epithelial cells, which indeed represent the first barrier for VacA in the gastric mucosa. However, it may rapidly pass this initial barrier. Another of *H. pylori*’s toxins, the cytotoxin associated antigen A (CagA) comes to its aid by associating with the scaffolding protein ZO-1 in epithelial cells and disrupting their barrier function [26], possibly allowing VacA access past the epithelial barrier. Having accessed the lamina propria, it can get into contact with many other cell types, especially cells of the immune system such as granulocytes, macrophages, dendritic cells (DCs), natural killer (NK) cells, and B and T cells. There, VacA interacts with DCs to reprogram them towards a tolerogenic phenotype and may efficiently inhibit proliferation of T lymphocytes, as shown under *in vitro* conditions [27,28]. In recent years, VacA research has extended towards its immunomodulatory effects. By migration of lymphocytes, monocytes, and granulocytes to the inflamed area in the gastric submucosa, the fight of *H. pylori* for survival and persistence begins. This can, unfortunately, worsen the disease state, as the adaptive immune response is known for its collateral damage by the production of cytokines from effector cells [29]. Due to *H. pylori*’s long co-evolution with humans, VacA was able to adapt to host defence mechanisms, allowing *H. pylori* to survive and thrive despite the presence of an acquired immune response. *H. pylori*’s effect on the host immune response, as a whole, is unconventional, as it shows both signs of being anti-inflammatory and pro-inflammatory. On the one hand, it inhibits B- and T-lymphocyte activation by various means; on the other, it induces mast cell migration and the production of pro-inflammatory cytokines and chemokines [4,30]. Infection stimulates a humoral response with the production of IgA and IgG antibodies against various *H. pylori* antigens, also against VacA. Although these latter antibodies may interfere with the vacuolating activity, antibodies in general do not seem to faze the bacteria much, as suggested by their presence in chronically infected patients [31].

## 4. VacA—T Cell Interaction, Internalization, and Formation of Acidic Vacuoles

It is known that in order to exert its many effects, VacA must be internalized via endocytosis. VacA–target binding has been associated with the larger p55 subunit, but it has also been shown that both the p33 and the p55 subunits are required for internalization [15]. Since the formation of vacuoles is largely dependent on VacA acid-activation, it is assumed that VacA needs to join with the membrane in a monomeric form before internalization [32]. However, the vacuolating effect of this toxin is dependent on oligomerized VacA, as elegantly demonstrated by Vinion-Dubiel *et al.* (1999), showing that the addition of a mutant toxin prevented vacuole formation in a dominant negative fashion. Interestingly, the pore formation was inhibited by the mutant VacA (non-vacuolating), which oligomerized with the wild-type VacA, forming non-functional pores, and hence prevented swelling and vacuolation [14]. To internalize, VacA must bind to the cell, and various receptors have been shown to bind to VacA among different cell types [33,34,35]. In human T cells, the deciding binding receptor is the integrin β2 subunit, CD18, as identified by Sewald *et al.* in 2008. Ricci *et al.* has already described that vacuolating activity was interrupted when lipid rafts were disabled in a membrane via a cholesterol-sequestering agent [36]. This highlighted the important role of cholesterol-rich lipid rafts for a productive interaction of VacA with the target cell membrane. Furthermore, the important plasma membrane sphingolipid sphingomyelin (SM) was identified to be essential for VacA toxin binding to the plasma membrane. Taking this information together with the fact that the internalization was dependent on certain members of the protein kinase C (PKC) family, Sewald *et al.* suggested a working model for the internalization of VacA into human T cells [37]. This model suggests that VacA endocytosis is achieved by VacA’s binding as a monomer to sphingomyelin, resulting in its recruitment to the uropod of the T-cell trailing edge, where it then binds to the integrin β2 subunit CD18, which is associated with lipid rafts in activated T cells. Here, the integrin heterodimer (CD18 and CD11a) and the Leukocyte function-associated antigen 1 (LFA-1) oligomerizes, leading to the phosphorylation of Thr_758_ of CD18’s cytoplasmic domain by PKCη or PKCζ. As a consequence, calpain removes talin from the CD18 cytoplasmic tail, allowing the adaptor protein 14-3-3 to bind to phosphorylated Thr_758_, which subsequently initiates the activation and recruitment of small GTPases Cdc42 and Rac-1 to enable actin rearrangements that lead to the endocytosis of VacA into the cell [37,38]. During or after endocytosis, VacA molecules oligomerize to form anion-selective channels that facilitate the influx of chloride ions, which leads to the swelling of the endosome. For these channels to form, the aforementioned hydrophobic region near the amino-terminal is essential [32], as shown by the inability of a mutant lacking this region, VacAΔ6-27, to form vacuoles [14]. These vacuoles show VacA in their membranes as well as in their interior [5,37]. Shortly after infection, these vacuoles also contain markers pertaining to early and late endosomes, Rab-5 and Rab-7, respectively, and LAMP-1 colocalization, a marker for lysosomes [37], even though VacA escapes lysosomal degradation [39]. At 24 h after infection, the vacuoles show only the late endo/lysosomal markers Rab-7 and LAMP-1, suggesting maturation from early to late endosomes [37,40]. As the endosomes swell forming VCVs, they need to recruit further membrane material to enable expansion without bursting. The origin of this material is still of some debate, but it was demonstrated that the VCVs co-localize with the SNARE protein syntaxin-7, which is involved in intracellular membrane fusions, suggesting that the vacuoles integrate other endosomes, lysosomes, or both upon vacuolation, enabling them to grow in size [41]. However, the VCVs apparently do not associate with the recycling endosomes, as indicated by the lack of VacA interaction with Rab-11 [37].

## 5. VCV Intracellular Transport, Toxin–Host Protein Interaction, and the Targeting of Cellular Organelles

**Figure 1:**
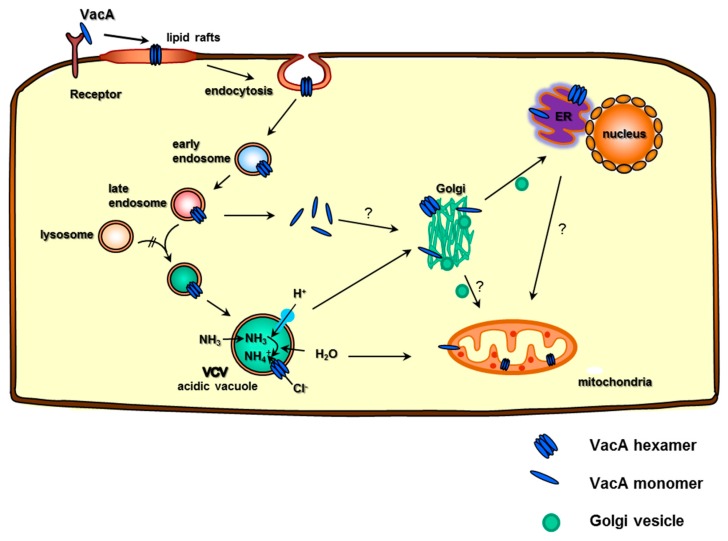
VCV intracellular transport, the toxin–host protein interaction, and the targeting of cellular organelles. VacA interacts with the host cell membrane binds to a cellular receptor and associates with lipid rafts. As a consequence, VacA enters into the membrane and oligomerizes to form anion-selective channels. Next, the VacA-receptor complex is endocytosed and matures from early via late endosomes into acidic vacuoles to form VacA-containing vacuoles (VCVs). The V-ATPase acidifies the endosomal compartment, resulting in the uptake of weak bases (NH_3_) and H_2_O. The VacA channel supports the entry of Cl^−^ ions, resulting in their swelling. VacA then might contact the Golgi apparatus either as a VCV or by entering the cytosol to bind to and pass the mitochondrial membranes by a yet unknown mechanism. Furthermore, VacA seems to also make contact with the ER.

The VCV mode of transport is still poorly understood, but it is known that they acquire an actin tail and use this to propel themselves around the cell cytoplasm [42]. It has long been implicated that VacA reaches mitochondria causing measurable effects [21,43] and has more recently been seen to co-localize with the ER and the Golgi [5]. It enters the cell in an endocytic vesicle that matures into a late endosome-like VacA-containing vacuole (VCV). Lysosome–endosome hybrids usually travel to the Golgi apparatus, where their content is redistributed around the cell in Golgi vesicles via the Trans Golgi Network (TGN). Since the VCVs seem to co-localize with the Golgi, VacA might contact the Golgi in this way. Thereby, VacA possibly uses various transport methods to get to its desired locations (Figure 1). 

**Figure 2:**
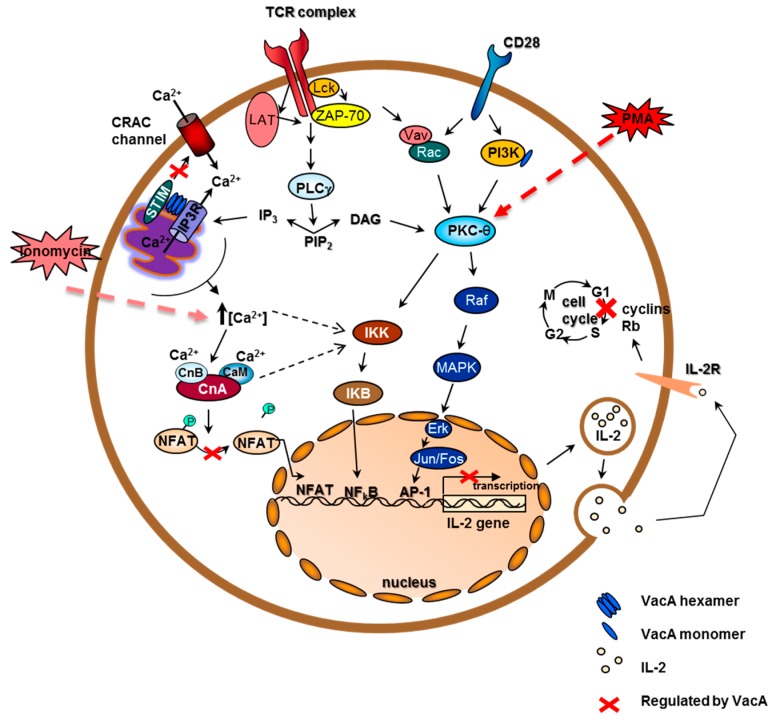
VacA interferes with T-cell activation and proliferation by manipulating the T-cell receptor pathway and the cell cycle. Upon the stimulation of T cells via TCR and CD28, two major pathways are activated (the Ca^2+^-dependent pathway and the MAP-kinase and NFkB pathway). The anion-selective channel activity of VacA is thought to depolarize the plasma membrane and to prevent the opening of the CRAC calcium channel, either directly or via the blocking of the ER-located Ca^2+^ sensor STIM1, which is operated by calcium released from intracellular stores. Alternatively, VacA might also block calcineurin activation directly. This might result in abrogation of transcription of IL-2 and IL-2Rα genes. At low doses, VacA inhibits T-cell activation by inducing a cascade of phosphorylation events involving a still unidentified receptor, Vav, and MKK3/6, resulting in an increase of the active form of p38. Vav induces actin rearrangement through the small GTPase Rac, which leads to inhibition of T-cell proliferation. Abbreviations: CRAC: Ca^2+^ release-activated Ca^2+^ channel; CD28: costimulatory molecule; CnA calcineurin A-subunit; CnB: calcineurin B subunit; CaM: calmodulin; TCR: T-cell receptor; NFAT: Nuclear Factor of Activated T cells; PKC: protein kinase C; PLCγ1: Phospholipase C γ1; IL-2: interleukin-2; IL-2R: Interleukin-2-Receptor; Rb: retinoblastoma protein.

VacA not only travels within the cell’s cytoplasm itself, it can also alter vesicular trafficking in cells [44,45,46] and can alter the protein content of endocytic compartments [40]. Recently, the contents of these VCVs were precisely analyzed to give an insight into their function [5]. VCVs were shown to contain 122 VCV-specific proteins coming from various cellular compartments, including 24.5% that are usually located at the nucleus, 12.3% in the mitochondria, 11% at the ER, and even 4.9% in the Golgi [5]. These proteins also had a wide range of functions, among which the signaling proteins made up a large part; however, interestingly, 14.7% of the proteins found were involved in vesicle trafficking. VacA was successfully identified to co-localize with the ER via fluorescence microscopy and via Western blotting and mass spectrometric analysis of the VCV (Figure 1) [5]. Interestingly, this study also identified lysosomal membrane proteins as well as ER markers, including membrane and luminal proteins, mitochondrial markers including inner membrane proteins, and Golgi markers, including membrane lipid anchors and vesicle proteins to be VCV-specific. Colocalization of VacA with the ER was demonstrated by fluorescence microscopy and validated by Western blotting and MS data. VCVs also contained the protein ITPR3, a calcium channel in the ER and STIM1, a calcium sensor in the ER (Figure 2). Interestingly, the calcium sensing, channeling, and signaling in the ER are very important for the activation of T cells *in vitro* and *in vivo*, and their presence in the VCV along with the fact that VacA inhibits T-cell activation is remarkable. The VCVs also contained other proteins involved in the immune response, totaling 6.3% of the VCV-specific proteins found, and this gives rise to further questions about VacA’s immunoregulatory activities [5].

## 6. Cellular Immune Response and Immunoregulatory Activities of VacA in Human T Cells

Very little is known about the immune response to *H. pylori* in an acute infection setting, as most patients are asymptomatic and infection often occurs in early childhood. However, chronic infection can be readily investigated [47]. Naïve CD4^+^ T cells can be differentiated into T helper 1 (Th1), Th2, Th17, and T regulatory (Treg) phenotypes, depending on the cytokine milieu with which they are confronted. Th1 cells can produce interleukin-2 (IL-2) and interferon-gamma (IFN-γ) and aid in cell-mediated immune responses, whereas Th2 cells can produce IL-4 and aid B cells to activate and differentiate. The only recently identified Th17 cells are often involved in host defence mechanisms involving extracellular bacteria. Tregs, on the other hand, are capable of suppressing these effector T cells in their cytokine production and proliferation and therefore act as a crucial part of the immune response to pathogens and regulating inflammation to prevent host damage [47]. In contrast, increased numbers of Tregs have been associated with peptic ulceration and gastric adenocarcinoma [48]. Thus, the ability of VacA to regulate the expression of Foxp3, a regulator of Tregs, and miR-155 was discovered [48]. In another study, Tregs marked as CD4^+^ /CD25^high^, were isolated from *H. pylori*-infected patients and were shown to induce memory T-cell suppression when added to memory cell assays [49]. (Further details on the VacA immunoregulatory activities pertaining to Tregs can be found in the accompanying review by Djekic and Müller). In the same study, the authors showed that this unresponsiveness is limited to *H. pylori*-specific memory cells, as there was no marked decrease in the response of memory cells to tetanus toxoid. However, in their setup, the addition of IL-2 was able to overcome the suppression.

The *H. pylori* T cells response is largely made up of Th1 and Th17 T helper cells [30]. As Th1 cells are producers of IFN-γ, a major risk factor in the development of gastritis, and IL-2, which is important for T-cell proliferation and hence bacterial clearance, a Th1 response usually increases gastritis severity and leads to lower colonization densities [30]. In vaccine studies in mice, successful vaccinations resulted in a shift from a Th1 to a Th2 response. A general increase in the Th1 response results in a stronger *H. pylori-*induced gastritis [50]. Curiously, vaccine-induced protection in IL-4 deficient mice suggests that a Th2-derived response is not needed for protection [51]. 

In humans, VacA shows mainly immunosuppressive tendencies towards T cells, it can inhibit IL-2 production and T-cell activation [9,27,28] and, in conjunction with the gammaglutamyl-transferase (GGT), promotes persistence and immune tolerance [52]. The VCV content strongly suggests an interaction of VacA, or VCVs, with many parts of the T-cell activation system as well as with other immune response molecules. 

A major effect of VacA on T cells is the inhibition of their proliferation, but also the suppression of IL-2 production and secretion. Several independent groups reported that VacA tested *in vivo* caused a marked decrease in IL-2 secretion in the human Jurkat T-cell line, which was artificially stimulated by phorbol myristate acetate (PMA) [9,27,28]. Two of these groups also demonstrated the effects of VacA on IL-2 production of primary human CD4^+^ T cells and found that it was relatively unchanged if the T cells were activated by anti-CD3 and anti-CD28 antibody activation (CD3/CD28 activation). Interestingly, IL-2 secretion of primary T cells treated with VacA was unchanged upon CD3/CD28 activation, but was markedly reduced upon PMA activation. This indicates that the inhibitory effect was downstream of the PMA activation pathway, which can be overridden by a TCR CD3/CD28 activation [28]. PMA induces NFκB via PKCθ to activate IL-2 transcription. The CD3/CD28 activation signaling utilizes many routes; in addition to signaling via PKCθ, it can also induce IL-2 production via IP3, causing a subsequent intracellular Ca^2+^ increase, which in turn activates NFAT transcription of the IL-2 gene, or it can even promote transcription via the AP-1 activator protein via diacylglycerol (DAG) signaling towards the Erk–MAPK–Jun/Fos pathway (Figure 2). 

*In vivo*, the T cells would be activated via their TCRs; hence, the CD3/CD28 activation is the more natural activation. The decrease in proliferation of primary human T cells or peripheral blood lymphocytes (PBLCs) treated with VacA is still very apparent [9,27,28]. IL-2 is needed as a proliferation stimulant by T cells, and evidence suggests that IL-2 secretion is relatively unchanged (in primary human CD4^+^ cells via CD3/CD28 activation) [28]. Surprisingly, proliferation is reduced despite IL-2 supplementation of the media, suggesting that the possible lack of IL-2, due to suppressive effects, was not the proliferation limiting factor [27,28]. It is implicated that T-cell proliferation inhibition is caused by inducing a cell cycle arrest in T lymphocytes [27,28], achieved by downregulating the cyclin D3 and E and consequently strongly reducing the phosphorylation of retinoblastoma protein (Rb), the downstream target of those cyclins [27]. It was demonstrated that proliferation was inhibited even if VacA was added 48 h after activating the cells, suggesting that VacA’s effect on proliferation is not through the inhibition of activation [28]. In 2007, Torres *et al.* demonstrated that VacA inhibits the proliferation of T cells that are stimulated by PMA/ionomycin or by CD3/CD28 activation, and of B cells stimulated either by PMA/anti-IgM or by T cells. This effect was evident in CD4^+^ and CD8^+^ T cells. The study went on to show that the proliferation of CD4^+^ T cells was also inhibited when the cells were activated by superantigens on antigen presenting cells (APCs) [53]. VacA suppresses the IL-2 production by various means. One of which is by interfering with the calcium signaling pathway at the level of the calcium-calmodulin-dependent phosphatase calcineurin [27], thereby preventing the nuclear translocation of nuclear factor of activated T cells (NFAT) and resulting in the downregulation of transcription of the IL-2 gene (Figure 2). Interestingly, VCVs contained IP3R and were associated with STIM1, both of which are involved in calcium signaling in the ER, but also identified some PKCs needed for T-cell activation and IL-2 production as well as PI3 kinase (PI3K), another T-cell activation factor. Thus, the VCV content strongly suggests an intensive interaction of VacA, with many components of the T-cell activation system as well as with other immune response molecules (Figure 2). 

## 7. Concluding Remarks

We have learned much about the structure and function of the vacuolating toxin of *H. pylori* over the last quarter of a century. We know about several components of the host cell membrane that are targeted for VacA internalization, and we know about some of the players involved in the endocytosis process. We have recently learned that VacA-containing vacuoles (VCVs) formed after VacA uptake into T lymphocytes are considerably complex membrane and protein assemblies that seem to target several host cell organelles when VacA travels through the host cell cytoplasm. However, we still do not understand the major strategies very well, how VacA induces its major immunomodulatory activities, neither in antigen presenting cells nor in T cells. Calcineurin was identified earlier as a major intracellular target of VacA, but we know that many more targets of the calcium signaling cascade of the T cells seem to be interaction partners of VacA in VCVs and therefore within the scope of VacA. In the future, we hope to sort out which of the VacA interaction partners are of relevance for the immunoregulatory activities of this fascinating bacterial toxin.
